# A Rare Case of Histopathologically Confirmed Creutzfeldt–Jakob Disease from Romania, Long Route to Diagnosis—Case Report and an Overview of the Romanian CJD Situation

**DOI:** 10.3390/jcm11164803

**Published:** 2022-08-17

**Authors:** Krisztina Kelemen, Attila Kövecsi, Laura Banias, Izolda Klára, István Mihály, Csilla Forró, József Attila Szász, Szabolcs Szatmári

**Affiliations:** 1 2nd Clinic of Neurology, County Emergency Clinical Hospital of Târgu Mures, 540136 Târgu Mures, Romania; 2 Faculty of Medicine, “George Emil Palade” University of Medicine, Pharmacy, Science and Technology of Târgu Mures, 540142 Târgu Mures, Romania; 3 Department of Pathology, County Emergency Clinical Hospital of Târgu Mures, 540136 Târgu Mures, Romania; 4 Department of Neurology, Emergency County Hospital of Miercurea Ciuc, 530173 Miercurea Ciuc, Romania; 5 Department of Neurology, Fogolyán Kristóf County Hospital, 520064 Sfantu Gheorghe, Romania

**Keywords:** Creutzfeldt–Jakob disease, Romanian CJD situation, international collaboration

## Abstract

Creutzfeldt–Jacob disease is a progressive and ultimately fatal disease, representing one of the most common forms of prion diseases. It is a rare pathology presenting with various symptomatology, and the fact that a definite diagnosis can be obtained solely by neuropathological techniques makes it hard to recognize and diagnose. Here we present the clinical and neuropathological features of a 72-year-old woman, who originally presented in a county hospital, then, along with the disease progression, got transferred to a university center in Romania, where CJD-specific tests are rarely performed, and ultimately was diagnosed with the help of international collaboration. The purpose of this case report and review is to summarize the Romanian CJD situation until the present day, to place the Romanian CJD epidemiology in an Eastern European context, and to highlight the diagnostic options and possibilities for clinical practitioners. We would also like to draw attention to the need for a national surveillance system. By presenting the patient’s route in Romania from the first presentation to diagnosis, we would like to emphasize the importance of interdisciplinary and international collaboration, by which we managed to cross the regional diagnostic boundaries and create a possible diagnostic pathway for future cases.

## 1. Introduction

Currently, there are relatively few neurodegenerative diseases that lead to rapidly progressive cognitive decline and ultimately to death. Such a malady is Creutzfeldt–Jakob disease (CJD), in which case most patients die within a year (usually 6 months) of symptom onset. CJD is caused by misfolded, transmissible proteinaceous particles, namely prions (PrP^Sc^).

The cruelty of this disease manifests in the fact that as a rare pathology, it is seldomly recognized, and the definite diagnosis can only be made from histopathological samples, in general after death at postmortem neuropathological investigations. At present, there is no available efficient and disease-specific therapy, and the clinician must use a laborious combination of characteristic neuropsychiatric symptoms, MRI and EEG findings, and, if possible, CSF biomarkers such as 14-3-3 or total Tau proteins to make the diagnosis [1], but these investigations are hardly accessible in Romanian University Centers, and even less so in smaller hospitals. Recently, RT-QuIC in CSF has proved to be a highly specific test for the detection of misfolded PrP, but it is not accessible for the suspected patients in Romania.

Prion diseases in humans have been studied for more than 70 years. Up to this point, they include different human diseases, namely (1) **Creutzfeldt–Jakob disease (CJD) and its different forms** (sporadic CJD (sCJD), genetic CJD (gCJD), iatrogenic CJD (iCJD), and variant CJD (vCJD)), (2) **variable protease sensitive prionopathy**, (3) **Gerstmann–Sträussler–Scheinker syndrome**, (4) **fatal familial (and sporadic) insomnia,** and (5) **kuru** [2,3]. Based on the supposed etiopathogenic process, they are classified into three major groups: sporadic, genetic, and acquired [4]. Sporadic is the most frequent form, occurring mostly in the elderly, the survival rate after diagnosis being only around 4 months. Genetic forms are connected to mutations in the prion protein gene (PRNP), and they include familial CJD, fatal familial insomnia, and Gerstmann–Sträussler–Scheinker syndrome, which account for approximately 10–15% of prion diseases. The acquired group comprises kuru (linked to cannibalism), iatrogenic CJD (after cadaver-derived dura mater grafts, growth hormone treatment, corneal grafts, and neurosurgery [5]), and variant CJD, respectively, which occurs at a younger age and has been related to the consumption of BSE-contaminated products.

Creutzfeldt–Jakob disease, also known as transmissible spongiform encephalopathy, has heterogeneous symptomatology. In the early stages of the disease, behavioral, emotional, and intellectual changes can be observed frequently in association with confusion, hallucination, and agitation. Cerebellar ataxia and visual disturbances may also occur [6]. Myoclonic contractions often appear within a few weeks, and they quickly become generalized. They can also be present until the akinetic-mute state, and finally, coma becomes dominant. Taken together, we can highlight the rapidly progressive dementia, cerebellar ataxia, and myoclonus as cardinal signs [1].

Around 90% of prion disease cases are represented by sporadic Creutzfeldt–Jakob disease [1]. The global incidence of CJD is reported to be 1–2 cases/million person/year, making it the most common form of human prion disease, but this number highly depends on the monitoring system, the local development of the diagnostic tools, and the knowledge of healthcare workers about prion diseases [4].

For example, EuroCJD is a network established in 1993 to conduct epidemiological surveillance for CJD. Romania is not part of this or any other surveillance system. Further information on the number of cases can be obtained by monitoring the case reports and studies. Uttley et al. reported unpublished data from the Euro CJD network obtained through personal communication, which showed a slight increase in deaths from probable or definite sporadic Creutzfeldt–Jakob disease between 1996 and 2018, in several countries, including Austria, Slovakia, and Slovenia [4].

According to the International Surveillance Network EuroCJD, during the period between 1993 and 2018, the incidence of sporadic CJD (which represents about 85% of CJD cases) was highest in Switzerland with 1.73 per million people, followed closely by France with 1.60 per million people, whereas the third place went to Austria with 1.52 per million people. The lowest incidence was in Estonia, with 0.32 per million people. The incidence and mortality in Central Europe are represented in Table 1.

The overall prevalence of prion diseases in Hungary is similar to that in other countries, but, interestingly, the risk of the genetic form is higher, as suggested by a report completed in 2005. This is supported by the high number of cases with E200K mutations and positive family history [7].

It is evident that the more precise and advanced the surveillance system is, like in Western Europe, the more cases are detected and confirmed.

Although there should be 1–2 cases/million person/year in Romania, in the last 15 years, only 10 cases have been reported, and exclusively in scientific papers as case reports. Out of these, only 2 were reported as confirmed cases with neuropathological techniques (Ceusu et al., 2015, and Corina Roman et al., 2013) [8,9]; the rest, including a small case series study by Andone et al. in the 1st Neurology Clinic, Mures County Emergency Hospital between 2007 and 2017 [10], were probable sCJD (2019 Catana et al., 2014 Cofoian-Amet et al., 2012 Pop et al.) [11,12,13]. All cases but one published in Romania were sporadic CJD, the exception being a 26-year-old female patient who reportedly had vCJD with psychotic behavior as the onset. The youngest patient with sporadic CJD was 36 years old, the oldest was 69 years old, and 70% of the patients were female. None of the patients suffered surgical interventions or other treatments known to be associated with the transmission of iatrogenic CJD. In all cases, the family history was negative for CJD and other neurodegenerative diseases. All patients died 3–4 months after hospitalization.

The low reported case number is an alarming result, and it is most probably due to the fact that confirmation of CJD from autopsy or brain biopsy is required to obtain a definitive sCJD diagnosis, and also due to low clinical ascertainment, autopsies are not routinely performed on patients with suspected CJD in Romania, and less so in the ‘COVID-times’, during which in general the number of performed autopsies has dropped significantly. Furthermore, in Romania, blood or organ donors are not specifically screened for CJD. If suspicion arises, then the person cannot become a donor.

In our country, the diagnosis of CJD faces many challenges. Most patients only have the clinical presentation and possible EEG and MRI findings to support the diagnosis, while central nervous system neuropathological investigations are limited. Furthermore, as it is a rare disease, the lack of experience in the diagnosis of CJD could also lead to misclassification as other neurodegenerative disorders. Several cases have been reported where CJD mimicked rapidly progressive neurological disorders such as encephalitis, dementia with Lewy bodies, Alzheimer’s disease, hyperparathyroidism, and even stroke [14,15,16,17,18].

With the help of international and interdisciplinary collaboration, we managed to cross the regional diagnostic boundaries and created a diagnostic pathway for future cases.

## 2. Case Presentation

We present a 72-year-old hypertensive woman, without other relevant medical histories (i.e., no tick bites or infections/febrile illnesses in recent history, without prior surgical interventions, no treatment with growth hormone or other treatments associated with iatrogenic CJD) and without previous cognitive impairment, who developed, from the end of December 2018, several episodes of spatial and temporal disorientation. In the following month, she developed signs of generalized rigidity and incomplete urinary retention. For this reason, an indwelling urinary catheter was inserted. Antiparkinsonian treatment (with levodopa and carbidopa) and anti-dementia treatment were initiated in January 2019 without any beneficial effect.

At the beginning of February 2019, she presented at the emergency department of a county hospital with rapid progressive aggravation of her symptoms, including aphasia and impaired level of consciousness. At the time of presentation, the patient was already immobilized.

Neurological examination showed a slight nuchal rigidity, normal cranial nerve function, and preserved motor strength, but extrapyramidal signs were present with increased rigidity. Reflexes were difficult to examine due to the rigidity, bilateral Babinski’s sign and palmomental reflex that were present. Sensory function, gait, and coordination were impossible to examine due to the altered mental status of the patient: Glasgow coma scale (GCS), 12 points (ocular response: 4, verbal response: 2, motor response: 6).

A cranial CT scan described only a few microinfarcts in the parietal lobe, which did not explain the patient’s symptomatology. Cerebrospinal fluid (CSF) examination (glucose, protein, white cell count, and bacteriological culture) showed the normal range. Laboratory tests were in the normal range, except for elevated C-reactive protein and complement component 4 (c4) as well as a urinary infection. The autoimmune and infectious screening was negative. An extensive panel was performed to search for paraneoplastic syndrome, including abdominal ultrasonography, thoracic x-ray, and carcinoembryonic antigen, all with negative results.

**Figure 1:**
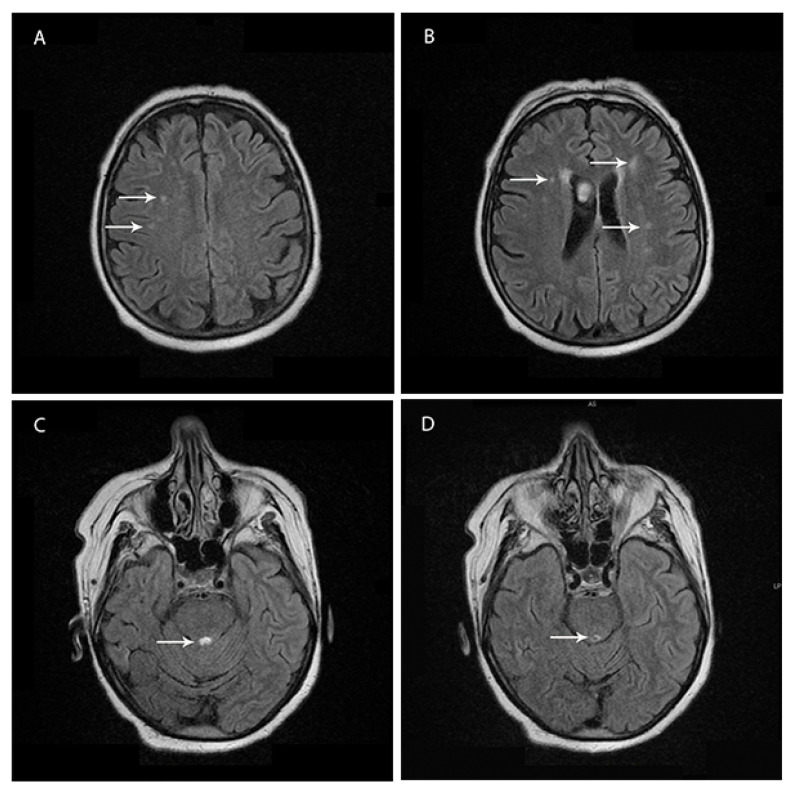
(**A**–**D**) Brain magnetic resonance imaging (MRI) study on T2-fluid attenuation-recovery (FLAIR). (**A**,**B**) Bilateral supratentorial subcortical increased signal intensities on FLAIR images. (**C**,**D**) Periaqueductal grey matter hyperintensity in FLAIR images.

Brain magnetic resonance imaging (1.5 T MRI—11 February 2019) showed bilateral and patchy supratentorial subcortical increased signal intensities on T2-fluid attenuation-recovery (FLAIR) images in frontotemporal regions and a demyelinating lesion surrounding the Sylvian aqueduct (Figure 1A–D).

During hospitalization, the patient received antiplatelet, prophylactic anticoagulant, antihypertensive, antibiotic, antiviral and gastroprotective medication.

As a consequence of the unfavorable evolution of the patient’s symptoms and the rapidly progressive character of the disease, she was transferred (12 February 2019) to the neurology clinic of a university teaching hospital to further investigate and elucidate the potential etiology.

Neurological examination at the moment of arrival showed increased nuchal rigidity without Kernig’s or Brudzinski’s sign, equal and reactive pupils, diminished gag reflex, spastic tetraparesis with the predominance of left hemiparesis, the spasticity was increased by tactile stimuli, including the eyelid; reflexes were brisk, and bilateral Babinski’s sign and palmomental reflex were present. To painful stimuli, she withdrew her limbs, but without localizing the pain, gait and coordination were impossible to examine due to the altered consciousness: GCS 9 points (O:1, V:2, M:6).

**Figure 2:**
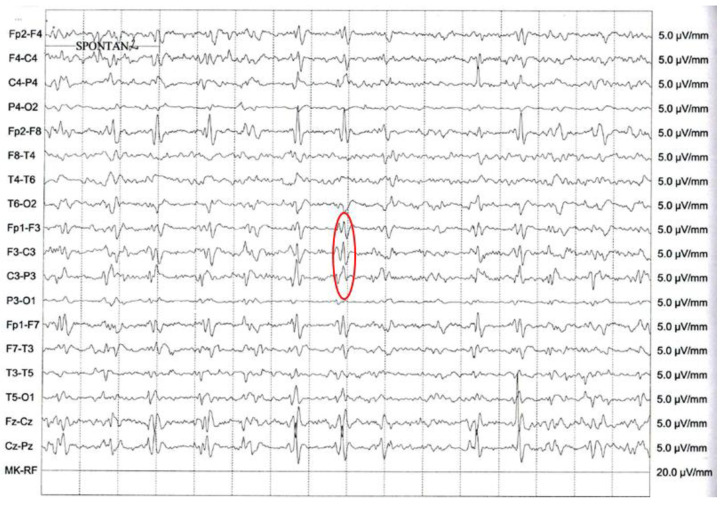
Periodic sharp wave complexes (PSWC) on the electroencephalography (EEG) recording of the patient. The red circle highlights the PSWC.

EEG (15 February 2019, performed approximately two months after the onset of symptomatology) showed diffuse slowing with frontal dominant irregular delta–theta activity. Pseudorhythmic, approximately 1 c/s, 200–400 ms, 50 µV bi- or triphasic sharp wave complexes, were seen over both hemispheres with fronto-central amplitude maximum (Figure 2). 

During the last hospitalization, the patient received antiplatelet, prophylactic anticoagulant, antihypertensive, lipid-lowering, antibiotic (ceftriaxonum and norfloxacinum), antiviral (aciclovirum), antimycotic (fluconazolum), gastroprotective, hepatoprotective, and cortico-therapy, neuro-roborant, and symptomatic treatment, respectively. Lumbar puncture was repeated to exclude autoimmune encephalitis, but the CSF examination was again within the normal range, and anti-NMDAR-antibodies were not present.

The patient’s condition progressively worsened; her state of consciousness sank to a deep coma (GCS 3 points), and 16 days after her admission (21 February 2019), she died of acute cardiorespiratory failure, after a total disease duration of roughly three months.

### 2.1. Neuropathological Examination

An autopsy was performed, with a suspected diagnosis of CJD, and the brain was fixed in 10% buffered formalin for three weeks. After sectioning, the brain tissues were immersed in 95% formic acid for 1 h to inactivate prion infectivity. The specimens were embedded in paraffin and cut into 3–4 μm sections, then deparaffinized, rehydrated, and stained with hematoxylin and eosin for routine pathological examinations.

For immunohistochemical analysis and confirmation of the diagnostic, three representative paraffin blocks were sent to the Division of Neuropathology and Reference Center for Human Prion diseases, Medical University of Vienna, Austria. The immunohistochemical analysis of selected sections was performed with anti-PrP antibody, alpha-Synuclein, βA4, and Tau-AT8, and is summarized in Table 2. The immunohistochemical analysis was performed by applying the anti-PrP 12F10 antibody (dilution 1:1000, epitope aa 142–160, CEA, Cedex, France) to the selected sections. Tissue pretreatment prior to PrP immunohistochemistry included 10 min hydrated autoclaving at 121 °C, 5 min, followed by immersion in 96% formic acid for 10 min at room temperature. The Dako Envision Kit (DAKO, Glostrup, Denmark) was used as a secondary system and diaminobenzidine as chromogen. For diagnosis, a negative control was included.

#### 2.1.1. Macroscopic Findings

The brain weighed 1145 g before fixation. Symmetrical mild atrophy of the cerebrum was observed. Coronal sections of the cerebral hemisphere revealed mild thinning of the neocortex without dilatation of the lateral ventricles. Substantia nigra presented normal pigmentation.

#### 2.1.2. Microscopic Findings

Sections from the cerebrum showed characteristic changes of spongiform encephalopathy. The frontal, cingulate and temporal cortex showed small vacuoles throughout the neuropil (spongiform change) associated with neuronal loss, diffuse astrogliosis, and microglial proliferation. Comparatively, the hippocampus was better preserved. The occipital cortex showed, in addition to small vacuoles, clusters of large confluent vacuoles.

#### 2.1.3. Immunohistochemical Findings

**Figure 3:**
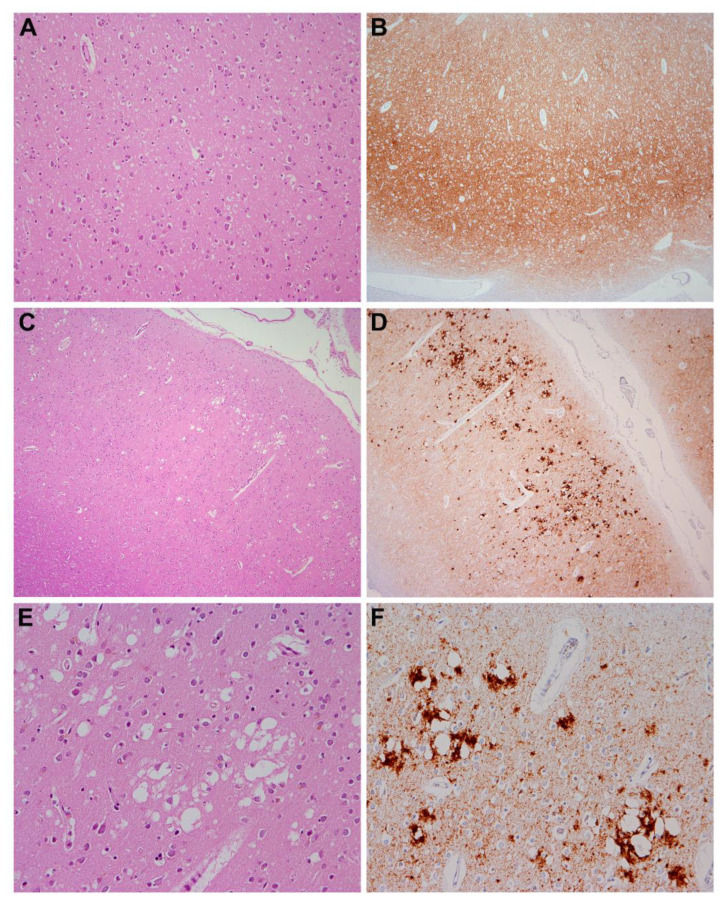
Representative microscopic findings (image kindly provided by Dr. Ellen Gelpi, Medical University of Vienna, Austria). (**A**) Mild spongiform change in cingulate gyrus consisting of small-sized vacuoles throughout the cortex; (**B**) immunohistochemistry showed widespread deposits of the pathological prion protein following a diffuse synaptic pattern; (**C**) in the occipital cortex, there were foci of large confluent vacuoles (enlarged in (**E**)); (**D**) immunohistochemistry revealed in these areas (occipital cortex) a patchy-perivacuolar deposition pattern of disease-associated PrP (enlarged in (**F**)). Original magnifications: (**A**,**F**): ×100, (**B**–**D**): ×40, (**E**): ×200.

Immunohistochemistry revealed the presence of pathological prion-protein deposits in a diffuse synaptic pattern in the cingulate, frontal, and hippocampus with a focal perineuronal pattern in the CA4 area of the hippocampus. In addition, and particularly in the occipital cortex, there were coarser deposits around confluent vacuoles following a patchy-perivacuolar pattern (Figure 3A–F). Furthermore, there was mild tau-positive neurofibrillary pathology in the hippocampal region with neurofibrillary tangles and neuropil threads in the CA1 area, in the entorhinal and transentorhinal region, corresponding at least to Braak stage II. There was no βA4-amyloid and no alpha-synuclein pathology in the hippocampus.

The morphological changes and the immunohistochemical features confirmed the diagnosis of Creutzfeldt–Jakob disease, suggesting a mixed MM1 + 2C histotype, according to Parchi et al. [19].

Due to technical and financial limitations, the patient was not genetically tested for codons 129 and 219.

## 3. Discussion

We present the clinical and neuropathological features of a patient with sporadic Creutzfeldt–Jakob disease, a rare condition, particularly in Romania, where CJD-specific tests are rarely performed. According to the European Creutzfeldt–Jakob Disease Surveillance Network diagnostic criteria for sCJD, a **possible sCJD diagnosis** should be considered when the patient shows rapidly progressive cognitive impairment combined with two of the following symptoms: myoclonus, visual or cerebellar impairment, pyramidal or extrapyramidal signs, and akinetic mutism. Additionally, the duration of the symptomatology must be shorter than 2 years. At the moment of the transfer between the two hospitals, the symptoms of our patient already pointed toward a possible diagnosis. In order to move on to a **probable sCJD** diagnosis, apart from the preexisting aforementioned progressive neuropsychiatric syndrome, at least one of the following should be documented: a typical EEG, a typical MRI brain scan, or a positive cerebrospinal fluid 14-3-3 protein test, or a positive real-time quaking-induced conversion (RT-QuIC—in cerebrospinal fluid or other tissues).

A typical EEG for CJD requires the presence of periodic sharp wave complexes, either simple sharp waves (biphasic or triphasic waves) or complexes with mixed spikes, polyspikes, and slower waves with a typical duration of 100–600 ms, recurring every 0.5–2 s [20]. The background signal regularly shows generalized low voltage slowing [21]. In the case of our patient, PSWC could be seen over both hemispheres approximately two months after the symptom onset. PSWC can be lateralized or generalized, tend to disappear during sleep, and are influenced by sedative medication. It has been observed that EEG shows characteristic changes depending on the stage of the disease, starting from nonspecific diffuse slowing and frontal rhythmic delta activity (FIRDA) in the beginning, and progressing toward periodic sharp wave complexes, finally leading to areactive coma traces *ante finem* [21].

A high signal in caudate/putamen on magnetic resonance imaging (MRI) brain scan or at least two cortical regions (temporal, parietal, or occipital) either on diffusion-weighted imaging (DWI) or fluid-attenuated inversion recovery (FLAIR), represent characteristic findings in patients with CJD. Less frequently, periaqueductal grey matter hyperintensity in FLAIR images has been reported, which was present on the MRI of our patient [22,23]. In recent years, the DWI sequence was reported as the most sensitive in CJD, and often the cortical diffusion restriction is the first clue in CJD [24]. Moreover, the disease progression can also be monitored using the DWI sequence [25]. In our case, the DWI sequence was without pathological changes, but the MRI was performed early on during the disease. In this phase of the diagnostic route, our patient could be classified as “probable” CJD. Unfortunately, in the case of our patient, the MRI could not be repeated in the later stage of the disease due to technical issues, as the patient’s deteriorated clinical state made it too difficult to perform an MRI.

Neither the testing of CSF for elevated levels of 14-3-3 protein nor the RT- QuIC could be performed due to technical limitations. To our knowledge, RT- QuIC cannot be assessed in Romania. Although protein 14-3-3 has 84% specificity and 94% sensitivity rate [26], there are only a few private labs that can determine the levels of protein 14-3-3 in Romania, and the high costs are often a burden to the patient’s family.

A **definite CJD** diagnosis can be made by the presence of a progressive neurological syndrome and either neuropathological, immunocytochemical, or biochemical confirmation. In our case, with the help of an international collaboration with the Division of Neuropathology of the Medical University of Vienna, Austria, the immunohistochemical detection of PrP^Sc^ could be performed and confirmed the suspected diagnosis. In order to determine the precise phenotype, and for definite diagnosis of **sCJD**, genetic testing of the prion protein gene (*PRNP*) should be performed, which was not available in our case.

The diagnosis of CJD faces many challenges but taking into consideration the nature of the disease, an accurate neuropathological diagnosis might provide closure to the family. As there is no current disease-modifying therapy or cure, the diagnosis could help provide appropriate palliative care and reduce the eventual risk of iatrogenic transmission. Establishing the diagnosis might be even more difficult when CJD coexists with other pathologies, such as Parkinson’s disease, Alzheimer’s disease, or Lewy body dementia [27,28]. Furthermore, by ruling out CJD, patients with mimicking conditions, such as autoimmune encephalitis, may receive appropriate treatment [29].

As in the case of other diseases that mainly affect the elderly, as a result of a globally aging population [30], a gradual increase in the incidence of sCJD should be seen locally as well. As we reported in previous papers in the case of patients living in nursing care in Romania, there are very limited data on the comorbidities, therapy, and course of the disease, and the autopsy findings are completely absent [31,32]. The reported incidence of CJD in Romania is surely an underestimate of the actual incidence of CJD-related cases and deaths, probably due to lack of awareness among clinicians, deficiency in diagnostic tools, and the absence of definitive and specific pathological examination of all cases.

As new diagnostic tools emerge, such as disease-specific protein aggregation and amplification assays, including real-time quaking-induced conversion (RT QuIC) [1], which could help early clinical confirmation, a definitive diagnosis is more easily reachable, but the accessibility of these tools should be improved in several countries.

By joining an international surveillance system, the visibility of CJD could be increased, and European/international diagnostic routes could be established to facilitate the diagnosis of CJD in smaller hospitals as well. The intensity and precision of the surveillance can influence the estimated incidence of this rare disease and help patient management. Another pertinent reason to set up surveillance in Romania is for vCJD, given its proximity to other countries affected by vCJD. Moreover, international collaboration facilitates the epidemiological comparison between nations, helps recognize atypical forms of prion diseases, and could also prevent the iatrogenic spread of the disease [29].

## Figures and Tables

**Table 1:** Incidence of CJD in some European countries (adapted from [4]).

Sporadic CJD	Period of Estimation	CJD Incidence or Mortality Per Million People
Hungary	1997–2018	1.07
Slovakia	1993–2018	0.86
Slovenia	1993–2018	1.46
Czech Republic	2000–2018	1.20
Greece	1997–2008	0.62
Italy	1993–2018	1.42
Germany	1993–2018	1.33

Creutzfeldt–Jakob disease (CJD).

**Table 2:** Summary of the antibodies used for the immunohistochemical analysis.

Antibody	Clone	Company	Dilution
anti-PrP	12F10, aa 142–160	CEA, Paris, France	1:1000
Tau-AT8	AT8, pS202/pT205	Thermo Scientific, Rockford, IL, USA	1:200
βA4	6F/3D	DAKO, Glostrup, Denmark	1:100
alpha-Synuclein	5G4	Roboscreen, Leipzig, Germany	1:4000

## Data Availability

The data presented in this study are available on request from the corresponding author. The data are not publicly available due to privacy reasons.

## References

[1] Hermann P., Appleby B., Brandel J.P., Caughey B., Collins S., Geschwind M.D., Green A., Haïk S., Kovacs G.G., Ladogana A. (2021). Biomarkers and diagnostic guidelines for sporadic Creutzfeldt-Jakob disease. Lancet Neurol..

[2] Prusiner S.B., Hsiao K.K. (1994). Human prion diseases. Ann. Neurol..

[3] Orrú C.D., Bongianni M., Tonoli G., Ferrari S., Hughson A.G., Groveman B.R., Fiorini M., Pocchiari M., Monaco S., Caughey B. (2014). A Test for Creutzfeldt–Jakob Disease Using Nasal Brushings. N. Engl. J. Med..

[4] Uttley L., Carroll C., Wong R., Hilton D.A., Stevenson M. (2020). Creutzfeldt-Jakob disease: A systematic review of global incidence, prevalence, infectivity, and incubation. Lancet Infect. Dis..

[5] Brown P., Brandel J.P., Sato T., Nakamura Y., MacKenzie J., Will R.G., Ladogana A., Pocchiari M., Leschek E.W., Schonberger L.B. (2012). Iatrogenic creutzfeldt-Jakob disease, final assessment. Emerg. Infect. Dis..

[6] Baldwin K.J., Correll C.M. (2019). Prion Disease. Semin. Neurol..

[7] Kovács G.G., Majtényi M. (2005). Creutzfeldt-Jakob disease in Hungary. Folia Neuropathol..

[8] Ceauşu M., Capatina C.O., Hostiuc S., Dermengiu D. (2015). A case of variant Creutzfeldt-Jakob disease in Romania. Rom. J. Leg. Med..

[9] Roman-Filip C., Ungureanu A., Filip D., Radu E., Zaharie I.S. (2013). Boala Creutzfeldt-Jakob sporadicǎ: Prezentare de caz şi discuţii asupra biomarkerilor diagnostici. Rev. Rom. Med. Lab..

[10] Andone S., Petrutiu S., Bajko Z., Motataianu A., Maier S., Macavei I., Stoian A., Balasa A., Balasa R. (2017). Sporadic Creutzfeldt-Jakob Disease: A clinical approach of a small case series and literature review. Rom. J. Neurol. Rev. Rom. Neurol..

[11] Catana M.G., Boiesan A., Roman-Filip I., Roman-Filip C. (2019). Sporadic Creutzfeldt Jakob disease with fast progressive evolution—A challenging diagnosis—A case report. J. Neurol. Sci..

[12] Cofoian-Amet Z., Rusu B., Mitu C., Rosianu E., Popescu B. (2014). Creuzfeldt-Jakob’s Disease-Case Report. Rom. J. Neurol. Rev. Rom. Neurol..

[13] Pop R., Teodorescu A., Tanasie M., Joikits R., Gheoca R., Simu M. (2012). A case of sporadic Creutzfeldt-Jakob Disease. Rom. J. Neurol. Rev. Rom. Neurol..

[14] Litzroth A., Cras P., De Vil B., Quoilin S. (2015). Overview and evaluation of 15 years of Creutzfeldt-Jakob disease surveillance in Belgium, 1998–2012. BMC Neurol..

[15] Krystina C., Abbas A., Hall A., Khadjooi K., Abasaeed-Elhag R., Rostami K. (2011). Sporadic Cjd Presenting With Aphasia Diagnosed in Medical Admissions Unit. Eur. J. Intern. Med..

[16] Pa̧chalska M., Kurzbauer H., Formińska-Kapuścik M., Urbanik A., Bierzyńska-Macyszyn G., Właszczuk P. (2007). Atypical features of dementia in a patient with Creutzfeldt-Jakob disease. Med. Sci. Monit..

[17] Hirst C.L. (2011). Sporadic Creutzfeldt-Jakob disease presenting as a stroke mimic. Br. J. Hosp. Med..

[18] Karatas H., Dericioglu N., Kursun O., Saygi S. (2007). Creutzfeldt-Jakob disease presenting as hyperparathyroidism and generalized tonic status epilepticus. Clin. EEG Neurosci..

[19] Parchi P., Giese A., Capellari S., Brown P., Schulz-Schaeffer W., Windl O., Zerr I., Budka H., Kopp N., Piccardo P. (1999). Classification of sporadic Creutzfeldt-Jakob disease based on molecular and phenotypic analysis of 300 subjects. Ann. Neurol..

[20] Gloor P. (1980). EEG characteristics in Creutzfeldt-Jakob disease. Ann. Neurol..

[21] Wieser H.G., Schindler K., Zumsteg D. (2006). EEG in Creutzfeldt-Jakob disease. Clin. Neurophysiol..

[22] Shiga Y., Miyazawa K., Sato S., Fukushima R., Shibuya S., Sato Y., Konno H., Doh-Ura K., Mugikura S., Tamura H. (2004). Diffusion-weighted MRI abnormalities as an early diagnostic marker for Creutzfeldt-Jakob disease. Neurology.

[23] Collie D.A., Sellar R.J., Zeidler M., Colchester A.C.F., Knight R., Will R.G. (2001). MRI of Creutzfeldt-Jakob disease: Imaging features and recommended MRI protocol. Clin. Radiol..

[24] Kumaran S.P., Gupta K., Pushpa B.T., Viswamitra S., Joshy E.V. (2012). Diffusion-weighted imaging: As the first diagnostic clue to Creutzfeldt Jacob disease. J. Neurosci. Rural Pract..

[25] Eisenmenger L., Porter M.C., Carswell C.J., Thompson A., Mead S., Rudge P., Collinge J., Brandner S., Jäger H.R., Hyare H. (2016). Evolution of diffusion-weighted magnetic resonance imaging signal abnormality in sporadic Creutzfeldt-jakob disease, with histopathological correlation. JAMA Neurol..

[26] Zerr I., Pocchiari M., Collins S., Brandel J.P., De Pedro Cuesta J., Knight R.S.G., Bernheimer H., Cardone F., Delasnerie-Lauprêtre N., Cuadrado Corrales N. (2000). Analysis of EEG and CSF 14-3-3 proteins as aids to the diagnosis of Creutzfeldt-Jakob disease. Neurology.

[27] Kovacs G.G., Alafuzoff I., Al-Sarraj S., Arzberger T., Bogdanovic N., Capellari S., Ferrer I., Gelpi E., Kövari V., Kretzschmar H. (2008). Mixed brain pathologies in dementia: The BrainNet Europe consortium experience. Dement. Geriatr. Cogn. Disord..

[28] Szász J.A., Orbán-Kis K., Constantin V.A., Péter C., Bíró I., Mihály I., Szegedi K., Balla A., Szatmári S. (2019). Therapeutic strategies in the early stages of Parkinson’s disease: A cross-sectional evaluation of 15 years’ experience with a large cohort of Romanian patients. Neuropsychiatr. Dis. Treat..

[29] Watson N., Brandel J.P., Green A., Hermann P., Ladogana A., Lindsay T., Mackenzie J., Pocchiari M., Smith C., Zerr I. (2021). The importance of ongoing international surveillance for Creutzfeldt–Jakob disease. Nat. Rev. Neurol..

[30] Szász J.A., Constantin V.A., Orbán-Kis K., Bancu L.A., Ciorba M., Mihály I., Nagy E.E., Szász R.M., Kelemen K., Simu M.A. (2021). Management challenges of severe, complex dyskinesia. Data from a large cohort of patients treated with levodopa-carbidopa intestinal gel for advanced parkinson’s disease. Brain Sci..

[31] Szász J.A., Constantin V.A., Orbán-Kis K., Rácz A., Arianabancu L., Georgescu D., Szederjesi J., Mihály I., Fárr A.M., Kelemen K. (2019). Profile of patients with advanced parkinson’s disease suitable for device-aided therapies: Restrospective data of a large cohort of romanian patients. Neuropsychiatr. Dis. Treat..

[32] Constantin V.A., Szász J.A., Orbán-Kis K., Rosca E.C., Popovici M., Cornea A., Bancu L.A., Ciorba M., Mihály I., Nagy E. (2020). Levodopa-carbidopa intestinal gel infusion therapy discontinuation: A ten-year retrospective analysis of 204 treated patients. Neuropsychiatr. Dis. Treat..

